# Mechanistic Insights into Biodegradable Silica–Starch Composite Materials—Structural and Adsorption Properties

**DOI:** 10.3390/ijms27146243

**Published:** 2026-07-14

**Authors:** Malgorzata Zienkiewicz-Strzalka, Magdalena Blachnio, Krystian Czuchryta, Anna Derylo-Marczewska

**Affiliations:** Department of Physical Chemistry, Institute of Chemical Sciences, Maria Curie-Sklodowska University, Maria Curie-Sklodowska Square 3, 20-031 Lublin, Poland; magdalena.blachnio@mail.umcs.pl (M.B.); krystiancz02@onet.pl (K.C.)

**Keywords:** silica–starch composites, biopolymer-based composites, porous materials, organic dyes, adsorption kinetics

## Abstract

Silica–starch composites were synthesized via a modified sol–gel route and evaluated as low-cost, biodegradable adsorbents for the removal of organic dyes from aqueous solutions. The formation of mesoporous hybrid networks and structural reorganization of starch upon integration with the silica phase were confirmed. The physicochemical properties of the composites were examined using nitrogen adsorption–desorption analysis, SEM imaging, SAXS, and XRD, providing complementary insights into their porous structure, morphology, and structural organization. Adsorption performance and kinetics were evaluated through continuous UV–Vis spectroscopic monitoring of dye concentration during the sorption process. Adsorption studies using methylene blue demonstrated that dye uptake proceeds through a combination of electrostatic interactions, hydrogen bonding between dye molecules and hydroxyl-rich starch domains, and diffusion-driven retention within the mesoporous silica framework. The proposed materials offer significant advantages arising from their simple, inexpensive synthesis and fully biodegradable nature. These features position silica–starch composites as sustainable sorbents suitable for environmentally oriented water purification applications.

## 1. Introduction

The increasing demand for sustainable and environmentally benign materials has driven intensive research toward hybrid composites combining inorganic matrices with natural biopolymers [1,2,3,4]. The biodegradability of biopolymers, leading to their decomposition into environmentally benign molecules, provides a promising alternative to conventional plastics, which pose an increasing environmental challenge [5,6,7,8]. Bionanocomposites combine ecological sustainability, inspiration from nature, and advanced functionality, forming the foundation of a new area of bioinspired hybrid materials [9,10,11,12]. Although various polysaccharides, including cellulose, chitosan, and alginate, have been successfully used for the preparation of sorption materials and possess a high density of hydroxyl groups [13,14,15], starch remains particularly attractive due to its low cost, widespread availability, biodegradability, facile processing, and ability to form hybrid composite structures with inorganic phases such as silica. Starch-based biomaterials are a growing area of interest, driven by the demand for sustainable and responsible materials and have recently even been referred to as bioplastics [16,17,18,19]. Among all possibilities, starch composites have attracted considerable attention due to their favorable physicochemical properties, low cost, renewability, and potential applications in environmental remediation [20]. Starch, as an available, renewable biopolymer composed of amylose and amylopectin units, is rich in hydroxyl functional groups which determine its ability to create gels, films, and composites with various mechanical and sorption properties. Surface functional groups provide multiple active sites for chemical interactions, including hydrogen bonding and coordination with adsorbates [4,21,22,23]. In recent decades, starch biocomposites and starch nanocomposites have been created by modifying the starch matrix with nanofillers such as aluminosilicate nanoparticles (e.g., montmorillonite [24,25,26,27,28,29]), metal oxide nanoparticles [30,31,32,33], and carbon nanostructures (graphene, graphene oxide, and reduced graphene oxide or carbon nanotubes) [34,35,36,37]. The incorporation of nanophases into the starch matrix leads to a significant improvement in the mechanical, thermal, and barrier properties of the materials, which expands their possible industrial and environmental applications through intense interfacial interactions [38,39,40]. Some examples include starch composites with layered silicates which exhibit increased tensile strength and better barrier properties, such as reducing water vapor permeability, which is desirable for packaging [41]. Modification with semiconducting metal oxides, in turn, has a positive effect on photocatalytic properties (both adsorption and degradation of dyes) [42,43,44], antibacterial activity [45,46,47], increased durability [48,49] of the material, and the possibility of sorbent regeneration under the influence of UV/Vis [50].

The integration of silica (both the amorphous and mesoporous silica phases) with starch enables the development of materials that merge the structural stability and high surface area of inorganic oxides with the biodegradability and functional versatility of polysaccharides [51,52,53]. The most commonly described strategy for the synthesis of starch–SiO_2_ nanocomposites is the sol–gel method, in which silica nanoparticles are generated in situ in the presence of a starch matrix. This process involves the hydrolysis of silicon alkoxylates (e.g., tetraethoxysilane) and the subsequent condensation of silanol groups, leading to the formation of a –Si–O–Si– network [54,55,56]. The presence of abundant hydroxyl groups in starch facilitates strong interfacial interactions with silica through silanol–polysaccharide interactions, resulting in mechanically stable hybrid networks [57]. The numerous –OH groups present in the starch structure enable intense interfacial interactions, primarily through hydrogen bonds, which promote the homogeneous dispersion of the nanophase, stabilization of the resulting hybrid structure, and uniform dispersion of the nanophase [54,58,59,60]. The literature emphasizes that starch in this system not only acts as a polymer matrix but also as a stabilizer for silica nanoparticle growth, limiting their agglomeration and enabling control of particle size at the nanometer scale [61,62,63,64,65]. An alternative approach to obtaining silica–starch nanocomposites is the physical incorporation of nanosilica into plasticized starch using mechanical or solution mixing methods. In such systems, the interactions between the components are primarily physical, which translates into lower interfacial stability compared to materials obtained via the sol–gel method [66]. However, even in such systems, an improvement in the mechanical and thermal properties of starch is observed, resulting from the strengthening effect of the nanophase and the limitation of the mobility of the polysaccharide chains. The presence of porous nanostructures increases the roughness and specific surface area of the material, which is important in the context of sorption applications, especially in the adsorption of cationic and anionic dyes [67] and other undesirable substances [68,69]. Several reports have highlighted the potential of starch–silica composites as adsorbents for pollutants from aqueous solutions [70,71]. In the context of pollutant adsorption, mesoporous silica nanophases promote the diffusion of dye molecules into the interior of the sorbent structure, increasing the adsorption capacity and process kinetics. For instance, starch–silica nanocomposites prepared using precipitation or sol–gel methods exhibited enhanced adsorption capacities toward organic dyes and heavy metal ions due to the synergistic effect of the developed surface area and polar functional groups [56,72,73].

The applications of silica–starch nanocomposites include sorbents for organic dyes such as methylene blue, methyl orange, rhodamine B, or Congo red and heavy metal ions. Adsorption on starch-based materials is governed primarily by the abundance of hydroxyl groups in amylose and amylopectin, which provide active sites for hydrogen bonding, dipole–dipole interactions, and coordination with polar or charged adsorbates [4,21,23]. In silica–starch composites, the adsorption mechanism becomes more complex due to the synergistic contribution of both phases. The silica network introduces mesoporous channels that facilitate intraparticle diffusion of dye molecules and increase the number of accessible adsorption sites [56,72,73]. Silanol groups (Si–OH) on the silica surface interact with dye molecules through electrostatic attraction, hydrogen bonding, and van der Waals forces, while the starch component enhances binding through its polar functional groups. For cationic dyes, adsorption is dominated by electrostatic interactions with negatively polarized oxygen atoms, complemented by multisite hydrogen bonding and physical entrapment within mesopores [74,75,76]. In the case of aromatic dyes, diffusion into mesopores and multipoint interactions with the sorbent surface also play a significant role. Numerous studies have demonstrated that starch-SiO_2_ nanocomposites exhibit higher adsorption capacity and better stability during adsorption–desorption cycles than unmodified starch. For example, in a study [77], a starch/silica nanocomposite achieved a very high maximum Cd^2+^ adsorption capacity (~769 mg/g) and maintained high efficiency over a number of adsorption–desorption cycles with only a nominal decrease in efficiency, showing better regeneration stability than unexpanded starch. In other studies, the starch-based nanocomposite showed good regeneration and stability: after 5 adsorption–desorption cycles, it retained >83% of its original efficiency [78]. It should be emphasized that even within starch adsorbents, very different experimental conditions and different kinetic models are used, making direct comparisons difficult. For example, studies on starch-halloysite composites, anionized starch–silica composites, or starch hydrogels report different equilibrium times, adsorption capacities, and kinetic parameters, even though the adsorbate is the same methylene blue [79]. Starch–silica systems are also being investigated as packaging materials and protective barriers. In the packaging sector, starch-based biocomposites are becoming a popular replacement for disposable plastic packaging. The addition of nanosilica improves the mechanical and barrier properties of starch materials, including reduced water vapor and gas permeability [80,81]. This effect is attributed to the extension of the diffusion path of molecules passing through the material and the limitation of the mobility of polysaccharide chains in the presence of a rigid nanophase. The literature also describes the use of starch–silica nanocomposites as carriers for active substances, including enzymes, drugs, and bioactive compounds. The porous structure of silica enables the immobilization of active molecules, while the starch matrix ensures biocompatibility and controlled release. Such systems have potential applications in biotechnology and biomedical engineering [82,83,84,85]. Furthermore, starch–SiO_2_ nanocomposites demonstrate potential as functional materials in heterogeneous catalysis and photocatalysis, particularly after further modification of the silica surface or the introduction of additional active nanophases [54,86,87,88]. The thermal and chemical stability of silica, combined with the easily modifiable structure of starch, enables the design of materials with tailored surface properties for specific technological applications.

Given the significant application importance of silica–starch composites, in-depth investigation of the interactions between adsorbates/bioactive substances/drugs and the adsorbent phases is essential, as well as an understanding of the mechanisms of processes occurring within the confined pore spaces. Studies of this type described in the literature require further experimental and theoretical investigations. Therefore, our research is focused on characterizing the composite properties and analyzing adsorption processes occurring within the internal pore space and the roles of various types of active sites. For various applications of porous materials, the adsorbate-adsorbent affinity and the adsorption rate are crucial factors, the combined effect of which determines the effectiveness of a given system. The obtained composite materials allow for the determination of the influence of structural and surface properties on adsorption processes occurring in the pores of materials with diverse phase compositions, which determines both the adsorption magnitude and its rate. This work presents the development and synthesis of starch–silica-based composites by a modified tetraethoxysilane hydrolysis-condensation procedure in an acidic environment, along with an assessment of the effect of colloidal silica content on the physicochemical properties of the obtained materials, as well as on the assessment of sorption properties towards organic dye substances based on experimental data of adsorption kinetics.

## 2. Results and Discussion

### 2.1. Textural, Structural, Morphological and Thermal Properties of Starch–Silica Composites

**Figure 1:**
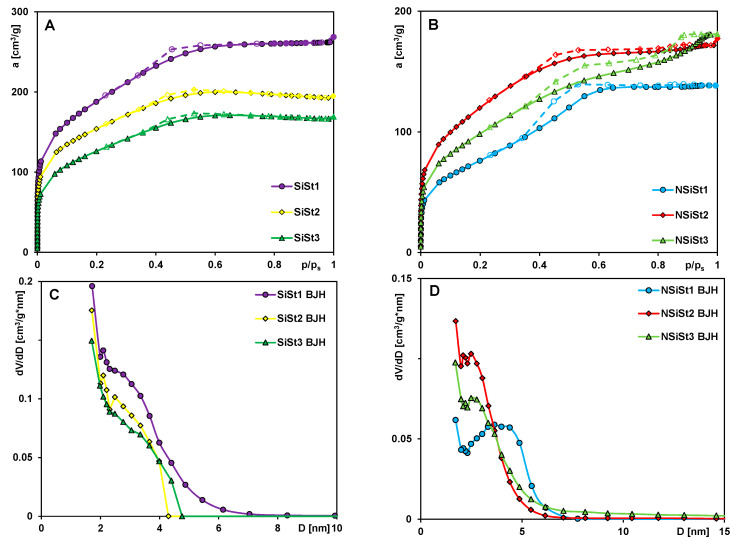

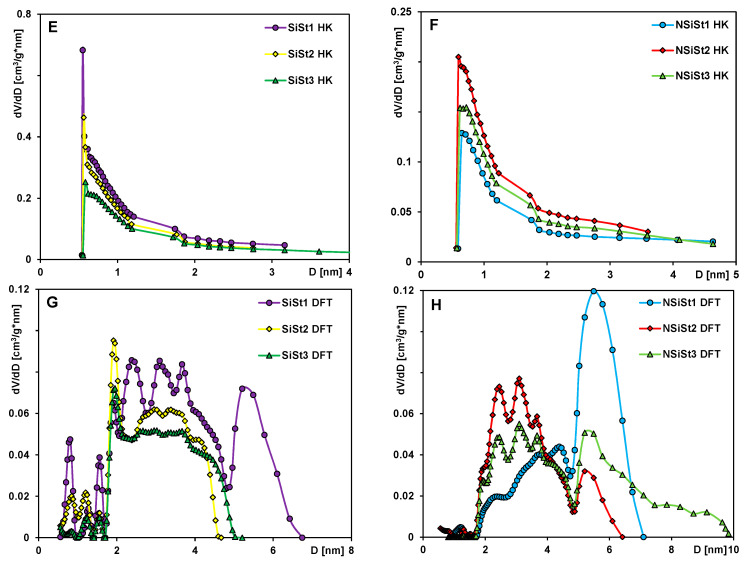
(**A**) Nitrogen adsorption–desorption isotherms for the starch–silica composites SiSt1–SiSt3 and (**B**) NSiSt1–NSiSt3, (**C**,**D**) BJH pore size distribution curves from the adsorption branch of isotherms for both groups of materials, (**E**,**F**) pore size distribution based on Horvath-Kawazoe (H-K) model, (**G**,**H**) Pore size distributions based on DFT calculations.

The experimental nitrogen adsorption–desorption isotherms recorded for the investigated starch–silica composites (exhibit a characteristic shape corresponding to type IV isotherms according to the IUPAC classification) clearly indicate the presence of a mesoporous structure (Figure 1A,B). The BET specific surface area values of the investigated starch–silica composites vary significantly depending on their composition and modification route (Table 1). Among the starch–silica materials (without nanosilica phase), the SiSt1 composite exhibits the highest surface area (676 m^2^/g), followed by SiSt2 (550 m^2^/g) and SiSt3 (455 m^2^/g). This indicates a gradual decrease in the accessible surface area with an increasing amount of starch and confirms its influence on the structural organization of the silica–biopolymer matrix. The incorporation of nanocolloidal silica leads to a noticeable modification of the textural properties. The NSiSt series shows lower values of specific surface area compared to the corresponding SiSt samples (S_BET_ = 277, 439, and 360 m^2^/g for the NSiSt1, NSiSt2, and NSiSt3 samples, respectively). This reduction in surface area can be attributed to partial pore blocking and aggregation effects associated with the presence of nanosilica, which may limit nitrogen accessibility to the smallest pores while simultaneously promoting the formation of larger mesopores.

A gradual increase in the amount of adsorbed nitrogen at intermediate and high relative pressures (*p*/*p*_0_ > 0.4), together with a distinct hysteresis loop, confirms the dominant contribution of mesopores in the studied materials. In the case of the SiSt1–SiSt3 samples, the observed hysteresis loop can be assigned to the H3/H4 type, suggesting the presence of pores with irregular geometry, such as slit-shaped interparticle voids formed as a result of silica particle aggregation and the incorporation of the starch matrix. The presence of nanosilica leads to a significant enhancement of the features associated with the developed mesoporous structure (Figure 1B). Two of these materials, NSiSt2 and NSiSt3, show similar total pore volume values to the SiSt2 and SiSt3 samples, but all are characterized by increased mesopore sizes (from 2.5 to 2.7 nm for SiSt1–SiSt3 samples to 3.2–3.3 nm for NSiSt1–NSiSt3). The hysteresis loop is wider and more pronounced, indicating greater pore heterogeneity and a more complex porous architecture compared to the samples without nanosilica. In the low relative pressure region (*p*/*p*_0_ < 0.1), a moderate increase in nitrogen uptake is observed, pointing to a limited contribution of micropores to the overall pore structure (only 5.2–7.3% of microporous surface area for NSiSt1–NSiSt3 and 20–27% of microporous surface area for SiSt1–SiSt3). These results indicate that the incorporation of starch into the silica matrix does not lead to a significant development of microporosity but instead promotes the formation of a mesoporous network with diverse pore geometry. This observation is also confirmed by the much wider and better visible peaks in the pore size distribution functions (Figure 1C,D, which were derived from the adsorption branch of the isotherms). Notably, the increase in pore size to approximately 5 nm should be attributed to the presence of nanocolloidal silica rather than to the biopolymer phase. The analysis of porosity in the smaller dimensions is somewhat different. Figure 1E,F shows the microporosity analysis obtained using the HK method, while Figure 1G,H shows the pore distributions determined by the DFT method using a cylindrical pore model for oxide surfaces. Systematic changes in the micropore size distribution curve for the samples with increasing amounts of starch indicate that these signals reflect the microporous structure of the biopolymer.

**Figure 2:**
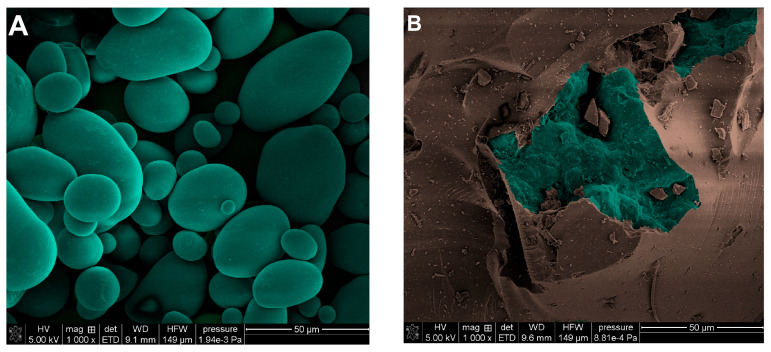

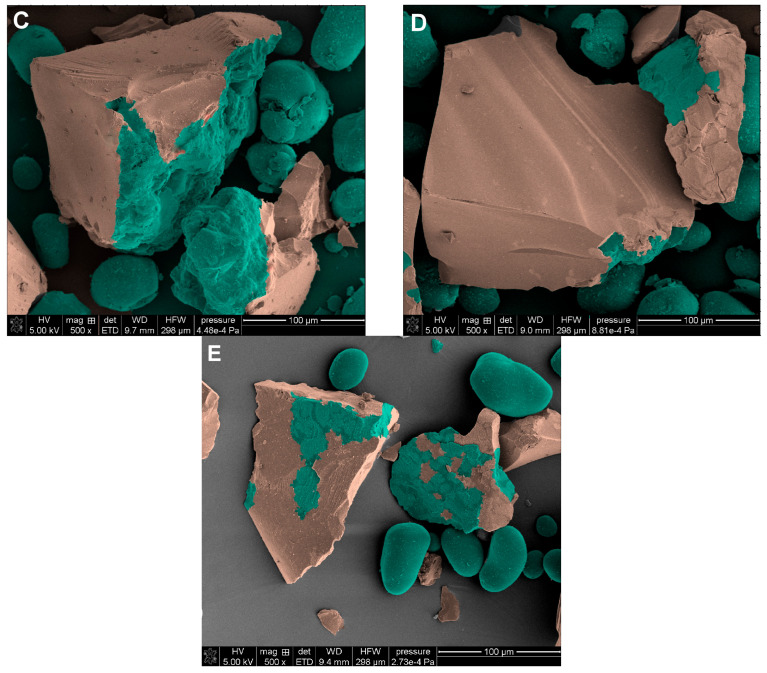
Scanning electron microscopy (SEM) images illustrating the morphology of the investigated starch and silica–starch materials: (**A**) SEM image of starch granules with predominantly oval and spherical shapes, (**B**) SiSt1 sample, (**C**) SiSt3, (**D**) NSiSt1, and (**E**) NSiSt3. The original SEM images are presented as Figure S2 in the Supplementary Materials File.

Scanning electron microscopy (SEM) images (Figure 2A) reveal the characteristic morphology of the native starch granules. The analyzed sample consists predominantly of granules exhibiting smooth surfaces and well-defined shapes, indicating the absence of significant mechanical or chemical damage during sample preparation. The granules display considerable diversity in size and morphology, which is typical for many botanical sources of starch. Most of the particles possess oval, ellipsoidal, or nearly spherical shapes, although elongated granules are also observed. The granule sizes can be estimated to range approximately from a few micrometers to several tens of micrometers, reflecting a polydisperse particle size distribution.

Scanning electron microscopy (SEM) observations reveal significant morphological differences between the native starch granules and the silica–starch composite with the lowest starch content (Figure 2B). The image shows that the originally well-defined granular morphology of starch is partially disrupted and embedded within a continuous inorganic matrix. Large fragments with irregular and fractured morphology dominate the observed microstructure. The surfaces appear rough, compact, and heterogeneous, which suggests the formation of a rigid composite structure resulting from the interaction between starch and the silica phase. In several areas, remnants of starch granules with partially preserved contours can still be distinguished; however, their surfaces are covered or surrounded by an additional phase attributed to silica. The highlighted region (green) corresponds to a silica-rich domain, which appears as a highly irregular, porous, and sponge-like structure. This morphology is characteristic of aggregated nanosilica particles forming a porous network. The presence of such domains indicates that silica forms continuous or semi-continuous clusters within the composite. The microstructure of the sample with a higher amount of starch (Figure 2C) is characterized by the coexistence of large irregular composite fragments and numerous intact starch granules, indicating a heterogeneous hybrid structure. Here, distinct starch granules with characteristic oval or spherical shapes are clearly visible in the surrounding regions. Many of these granules retain their original morphology, although some show surface deformation, partial collapse, or cracking, which may result from mechanical stresses during drying or composite formation. In the case of the second group of materials, the presence of fine grains of colloidal silica involved in the synthesis on the surface of the starch grains was additionally revealed (Figure 2D,E). SEM images for the remaining samples are presented in the Supplementary Materials File as a continuation of Figure S2.

**Figure 3:**
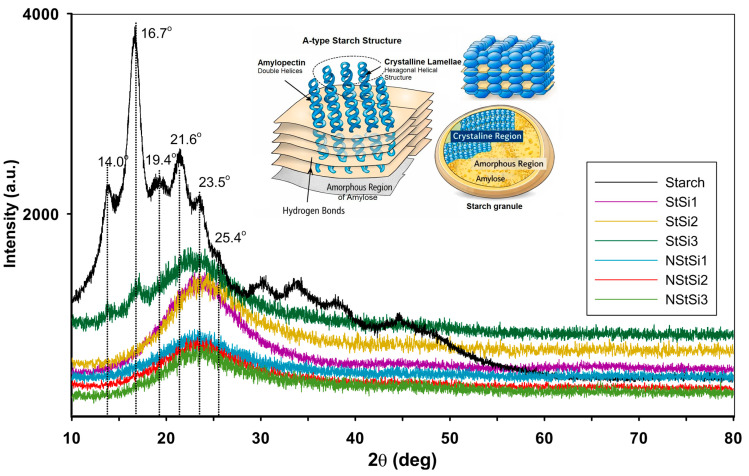
X-ray powder diffraction (XRD) patterns of silica–starch composites. For comparison, the X-ray powder diffraction pattern of the initial starch sample was also added.

Starch is a polysaccharide with variable amylose and amylopectin content, depending on the plant source. X-ray diffraction (XRD) analysis allows for the distinction of three basic starch crystallization types: A, B, and C, depending on the packing of polysaccharide chains into semicrystalline domains [89,90,91]. This classification is determined by the varying lengths of the amylopectin side chains and the degree of double-helical arrangement in the polysaccharide structure. The XRD patterns of the initial starch and starch–silica composites are presented in Figure 3. The diffraction profile of the unmodified starch exhibits distinct reflections at approximately 14.0°, 16.7°, 19.4°, 21.6°, 23.5°, and further signals in the range of 45° of 2θ, which are characteristic of the B-type for tuber and amylose-rich starches of hexagonal crystal structure [92,93] (inset in Figure 3). This crystallographic form is typical of cereal starches and is associated with the hexagonal packing of amylopectin double helices within the crystalline lamellae of starch granules. The relatively sharp and intense peaks indicate a well-developed semicrystalline structure composed of ordered crystalline domains embedded within an amorphous matrix.

In contrast, the XRD curves of the starch/silica composites display significant modifications in both intensity and profile shape. With one exception (the StSi3 sample), diffraction peaks characteristic of starch at 2θ = 14° and 16.7° are no longer clearly visible. In the case of the StSi3 sample, the highest starch content was used, which may indicate partial preservation of the original starch structure. The relatively high starch fraction suggests that not all polymer chains are completely integrated with the silica phase, allowing some crystalline domains to remain detectable. The observed structural modifications can be attributed to intermolecular interactions between the hydroxyl groups of starch and the silanol (Si–OH) groups on the silica surface. These interactions likely interfere with the native hydrogen-bonding network responsible for stabilizing the crystalline lamellae of starch, leading to partial amorphization of the material. Nevertheless, the persistence of characteristic A-type reflections indicates that the fundamental polymorphic structure of starch is preserved, albeit with reduced crystallinity (as for the StSi3 sample). A prominent feature observed in the powder diffraction patterns for both series of materials is a broad singlet in the 15–30° (2θ) range, associated with the amorphous nature of the silica phase. This broad halo indicates the incorporation of the biopolymeric phase into the inorganic matrix and a decrease in long-range molecular order resulting from the reduced relative crystallinity of the biopolymer phase. These structural changes suggest the partial disruption of the ordered amylopectin double-helical arrangement upon the incorporation of silica.

Differences in X-ray scattering intensity were observed among the samples with varying contents of both the polysaccharide and silica phases. In the case of the StSi1-StSi3 series, obtained using increasing amounts of the polysaccharide phase, the highest scattering intensity was observed for the StSi3 sample, while the lowest intensity was recorded for StSi1 (containing the lowest starch content). In this case, the polysaccharide component responsible for X-ray scattering likely exists in a nanostructured form with dimensions within the resolution limit of the measurement system, i.e., in the range of 1–100 nm. In the NStSi1–NStSi3 series, an increase in SAXS intensity was observed for the NStSi3 sample, which was prepared using the highest amount of the silica phase (500 mg). It should be noted that all samples in this series were synthesized with the same polysaccharide content and progressively increasing amounts of colloidal silica. Therefore, the enhanced SAXS signal for this group can be attributed to the presence of colloidal silica agglomerates.

Analysis of the shape of the SAXS curves recorded for the silica–starch samples reveals the absence of sharp oscillations over the entire measured range. According to literature reports, the intensity and clarity of this maximum may depend on the dispersion medium and the degree of hydration of the starch granule, which influences the electron density contrast between crystalline and amorphous regions [94,95,96]. In the case of silica–starch materials, the nature of this signal results from the interaction between the silica phase (also in the form of a silica precursor) and the polysaccharide material. Therefore, these signals are less pronounced and not as well separated as in the literature mentioned above. In the scattering vector range q = 0.03–0.08 Å^−1^, SAXS curves of starch exhibit characteristic intensity changes associated with the presence of an ordered lamellar structure within the starch granule. A broad diffraction signal is observed in this region, with a maximum occurring in the q range from 0.07 to 0.08 Å^−1^. This maximum is attributed to the periodic organization of alternating crystalline and amorphous lamellae in the amylopectin structure [97]. According to the analysis presented here [98], the SAXS maximum in this range corresponds to a characteristic structural distance of 9–10 nm, calculated based on the relationship *d* = 2*π*/*q*. This value reflects the periodicity of the lamellar arrangement in the starch granule, resulting from the alternating occurrence of ordered crystalline regions and less ordered amorphous domains. The signal observed at lower values of q ≈ 0.03–0.06 Å^−1^ may be related to scattering from larger structural heterogeneities, such as lamellae aggregates or mesostructural domains within the granule. However, an interference maximum may appear near q ≈ 0.09 Å^−1^, which also signals the presence of an ordered lamellar structure of starch.

**Figure 4:**
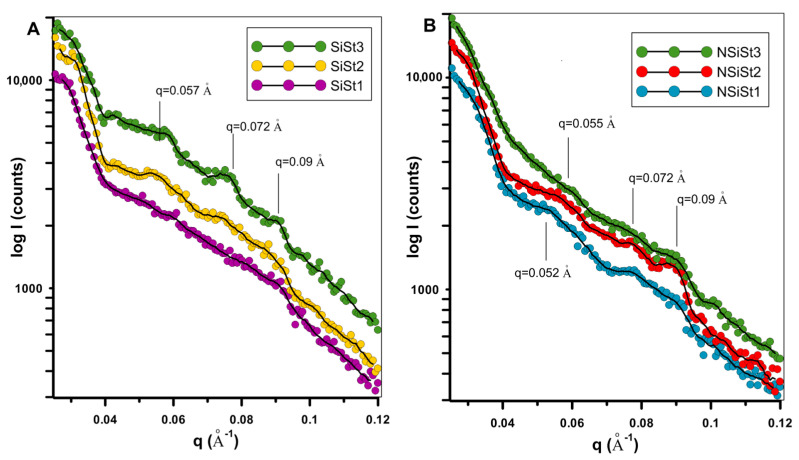
Experimental SAXS curves of starch–silica composites samples for (**A**) SiSt1–SiSt3 samples, and for (**B**) NSiSt1–NSiSt3 samples.

To characterize the nanostructural morphology of the silica-modified starch composites, the volume particle size distribution analysis (Dv(R)) based on small-angle X-ray scattering (SAXS) data (Figure 4) was applied. The obtained Dv(R) curve represents the inverse Fourier transform of the SAXS data and provides information on the spatial distribution of particles on the nanometer scale in terms of volume fraction. The Dv(R) function enables quantitative assessment of nanostructure sizes and the monitoring of morphological changes depending on the composite composition and processing conditions. In this case, Dv(R) analysis allows for the assessment of the dispersion quality of the biopolymer phase in silica–starch composites and the identification of the presence of macromolecular structures, which is crucial for the rheological, barrier, and mechanical properties of biodegradable materials.

**Figure 5:**
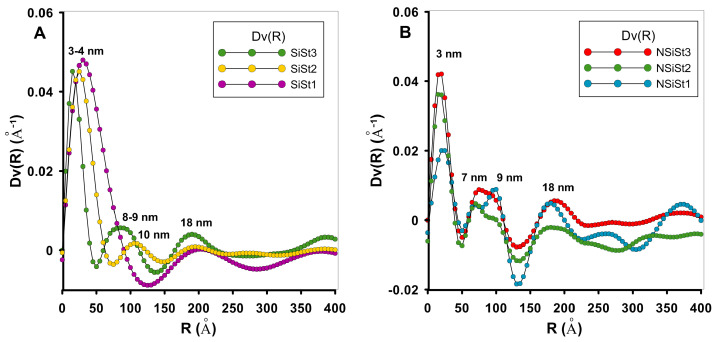
Dv(R) graphs showing the volume distribution of particles versus their radius R in the silica–starch composite, obtained from SAXS analysis for (**A**) SiSt1–SiSt3 samples, and for (**B**) NSiSt1–NSiSt3 samples.

The complexity of the obtained volumetric distribution (Figure 5) indicates the existence of a diverse particle population, which may be a consequence of the inhomogeneous dispersion of the biopolymer phase in the inorganic (silica) matrix.

The strongest signal for all samples appears in the 3–4 nm range, indicating the presence of very fine structural heterogeneities. In silica–starch composites, this signal can be attributed primarily to primary silica particles or very small silica aggregates that form the inorganic framework of the material. The intensity of this peak was almost constant for SiSt1–SiSt3 and decreased with increasing starch content for NStSi1–NStSi3 samples, suggesting partial masking of the silica electron contrast by the polysaccharide phase. The second maximum appears in the 8–10 nm range, which is comparable to the characteristic periodicity of the starch lamellar structure (approximately 9–10 nm), known from SAXS studies of starch. Therefore, in the composites, this signal can be attributed to fragments of amylopectin lamellar structures. As the starch content increases, this maximum becomes more pronounced, indicating a growing proportion of structures of this size. The presence of additional peaks located around 18 nm suggests the possible formation of particle aggregates, which may result from intermolecular interactions or insufficient stabilization of the system during mixing or gelation.

**Figure 6:**
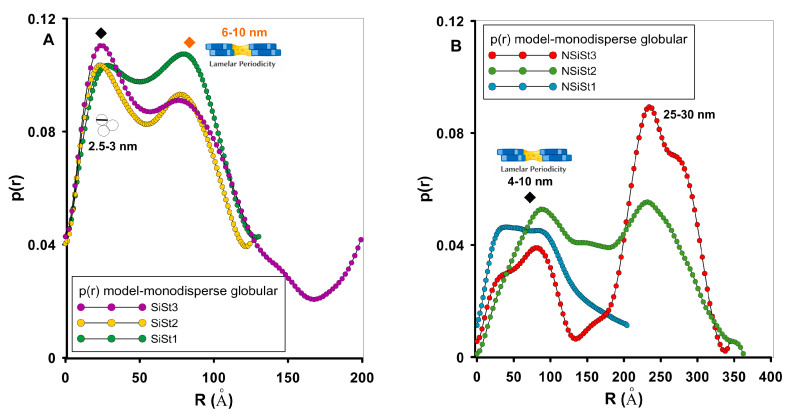
Functions *p*(*r*) for the obtained silica–starch materials for (**A**) SiSt1–SiSt3 samples, and for (**B**) NSiSt1–NSiSt3 samples.

The probability of specific distances between pairs of scattering points within a single particle or aggregate was analyzed using the pair distance distribution function (PDDF), denoted as *p*(*r*) (Figure 6). The PDDF is an intermediate result obtained by transforming SAXS data from the scattering vector space (q) to real space (R), typically performed using an indirect Fourier transform. The *p*(*r*) curve provides detailed information about the shape, size, and internal organization of the scattering particle. The position of the *p*(*r*) maximum indicates the most probable distance between points within the particle. Analysis of the symmetry and overall shape of the *p*(*r*) function also allows for the assessment of the scatterers’ morphology. Two characteristic maxima are visible in all curves, indicating the presence of two dominant correlation lengths in the materials’ structure. The first maximum, ~3 nm, corresponds to the most likely distance between the original electron contrast heterogeneities. In starch–silica composites, this can be attributed to primary silica particles or very small silica aggregates stabilized by starch. This maximum becomes more pronounced for the sample with the lowest starch content and decreases systematically with increasing starch content for the SiSt3 sample. The second maximum occurs in the range R ≈ 6–10 nm and corresponds to larger structural units that may be fragments of starch lamellar structures. In starch-containing materials, this length is often related to the organization of amylopectin segments, and this maximum is most pronounced for SiSt3, the material with the highest starch content. In this range, all samples exhibit a first maximum of the *p*(*r*) function corresponding to the periodicity of the starch lamellar structure, resulting from the alternating arrangement of amylopectin crystalline lamellae and amorphous regions, as observed for the first group of materials. This maximum is most pronounced for NSiSt1, and its intensity decreases with increasing silica content. This indicates that the addition of silica partially disrupts the local lamellar structure of starch. The second distinct maximum appears in the 25–30 nm range and is particularly intense for the NSiSt3 sample, which contains the highest amount of silica. Therefore, with increasing silica content, the contribution of the starch lamellar signal decreases (4–10 nm), while the intensity of the silica signal increases (~25–30 nm), and the composite structure transitions from a dominant polysaccharide structure to a structure controlled by the dispersion of SiO_2_ nanoparticles. Moreover, the separation of the two bands (between 10 and 20 nm) indicates little interaction or overlap between the populations of particles of different sizes, which demonstrates the complex nature of the internal structure of the silica–starch composite with colloidal silica.

**Figure 7:**
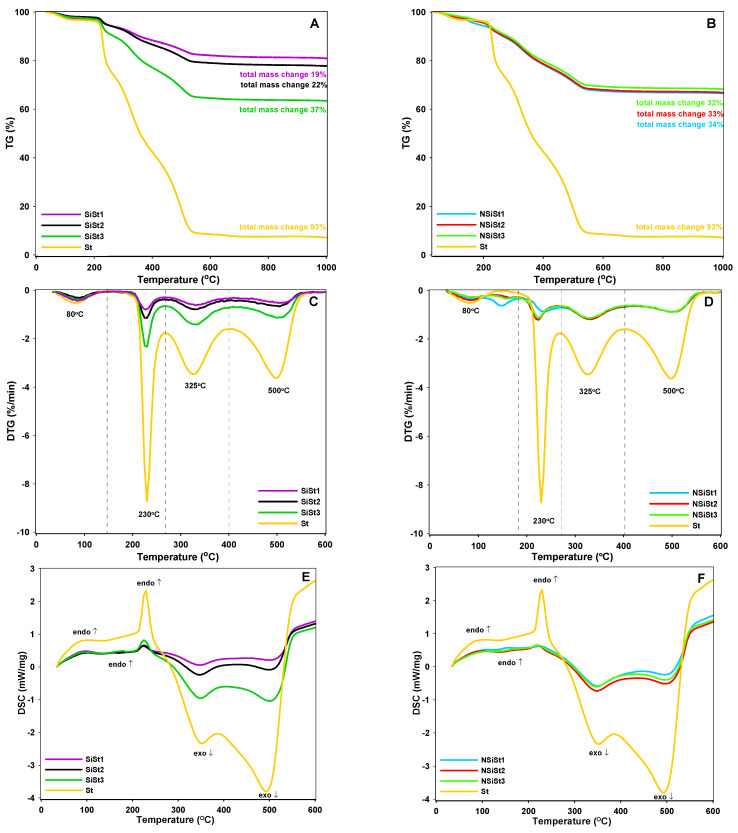
(**A**,**B**) TG, (**C**,**D**) DTG, and (**E**,**F**) DSC curves for neat starch (St) and the composites from the SiSt series (**A**,**C**,**E**) and the NSiSt series (**B**,**D**,**F**) measured under air atmosphere.

The TG/DTG/DSC analysis (Figure 7A–F) performed under air atmosphere indicates that neat starch and the silica–starch composites exhibit very similar thermal degradation profiles in terms of the characteristic temperatures of their thermal events. For all materials, the first mass loss step below 150 °C corresponds to the removal of physically adsorbed moisture. The associated DTG peak maxima (80–88 °C) and the weak endothermic DSC effect are consistent with dehydration processes involving hydrogen-bonded water. For the composites, an additional small peak is observed at approximately 150 or 160 °C, which can be attributed to the disruption of interactions between the silanol and hydroxyl groups. The second mass loss step, characterized by a distinct DTG peak in the range of 150–270 °C with a maximum at ~230 °C, corresponds to the initial starch depolymerization. This stage is associated with the cleavage of the most labile α-(1 → 4) glycosidic bonds in amorphous regions, as well as intramolecular dehydration reactions. The corresponding DSC signal is endothermic, reflecting the energy required for bond cleavage and structural rearrangement. The main degradation step with a relatively large mass loss occurs between approximately 260 and 400 °C, with DTG maxima at 325 °C for neat starch and 328–335 °C for the composites. The observed DTG T_max_ is consistent with literature data for starch decomposition in air [99,100]. This stage is accompanied by an exothermic DSC peak. Although depolymerization itself is endothermic, the overlap with oxidative reactions of carbonaceous structures results in an exothermic thermal effect. At higher temperatures (400–560 °C), a second exothermic DSC peak is observed, corresponding to the oxidative degradation and combustion of the carbonaceous residue. For starch, the DTG peak maximum appears at a temperature of 500 °C, whereas for the composites, it appears in the range of 499–507 °C. The detailed data for each degradation stage are summarized in Table 2. Analyzing total mass change in the studied materials, one can observe that neat starch undergoes almost complete mineralization, while the composites retain a substantial residual mass at elevated temperatures. Similarly, the subsequent mass losses, the rates of mass change, and the corresponding DTG and DSC peak intensities are slightly different between the samples. These differences are attributed to the presence of the thermally stable inorganic silica phase in the composites, which reduces the fraction of degradable organic material and increases the residual mass at high temperatures. The small difference in DTG T_max_ between the starch and composites falls within the typical experimental variation for such systems and suggests that the intrinsic thermal stability of the starch phase remains essentially unchanged after incorporation into the silica matrix.

**Figure 8:**
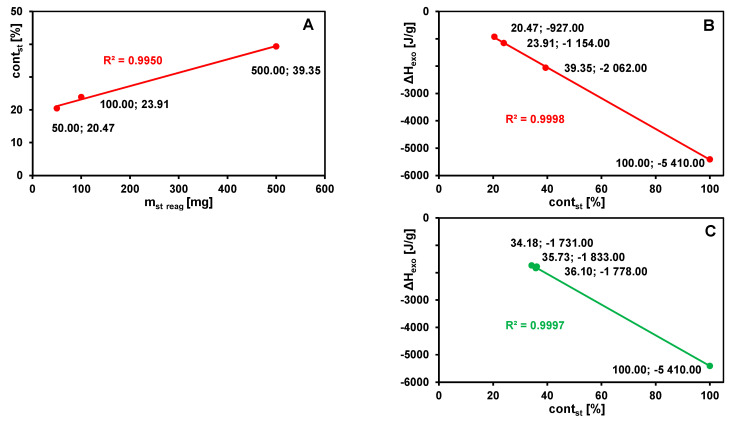
(**A**) Dependence of actual starch content in samples on amount of reagent used in the synthesis. Dependence of the specific enthalpy change in the exothermic processes on actual starch content in the composites from the SiSt series (**B**) and the NSiSt series (**C**).

Based on the total mass loss (Δm) measured for neat starch and the composites, the organic fraction in the resulting materials was quantified. In the SiSt series, the organic content reaches 20.47%, 23.91%, and 39.35% for SiSt1, SiSt2, and SiSt3, respectively. A comparison of these values with the amount of starch used in the synthesis of the individual composites reveals a very strong correlation (R^2^ = 0.9950), confirming that the actual composition closely reflects the nominal formulation (Figure 8A). A similar observation applies to the NSiSt series (34.18–36.10% of starch content), for which identical amounts of the organic reagent were used during synthesis. Figure 8B,C present the dependence of the specific enthalpy change in the exothermic processes (ΔH_exo_) (determined from the integrated areas under the third and fourth DSC peaks) on the starch content in the samples. The strong correlations (R^2^ = 0.9998 and 0.9997) between the energetic effects recorded during high-temperature treatment and the starch fraction in materials from the SiSt and NSiSt series, respectively, indicate that the inorganic phase (silica) has a negligible contribution to the observed transformations.

### 2.2. Kinetics of Methylene Blue Adsorption on Silica–Starch Composites

The obtained silica–starch composites were evaluated for their applicability as adsorbents for the removal of dyes from water and wastewater by conducting an in-depth analysis of the efficiency of the process, as well as the relationship between adsorption uptake and rate, adsorbent properties, and adsorbate–adsorbent interactions. Methylene blue was employed as a model adsorbate (molecular weight: 319.85 g·mol^−1^; pK_a_: 2.6 and 11.2; water solubility: 4%), while neat starch and silica were used as reference materials. The adsorption studies were primarily focused on kinetic aspects, enabling the assessment of both the rate of the process and the degree of water purification. In industrial treatment systems, adsorption processes often do not reach equilibrium due to the limited contact time between the adsorbent and the adsorbate. Consequently, the purification effectiveness is largely governed by the rate of pollutant uptake rather than by the adsorption capacity of the adsorbent determined from equilibrium isotherms. Moreover, kinetic analysis provides insights into the rate-controlling steps of the process, including diffusion from the bulk solution to the adsorbent surface, intraparticle diffusion within the pore structure, and the actual binding of molecules at active sites.

**Figure 9:**
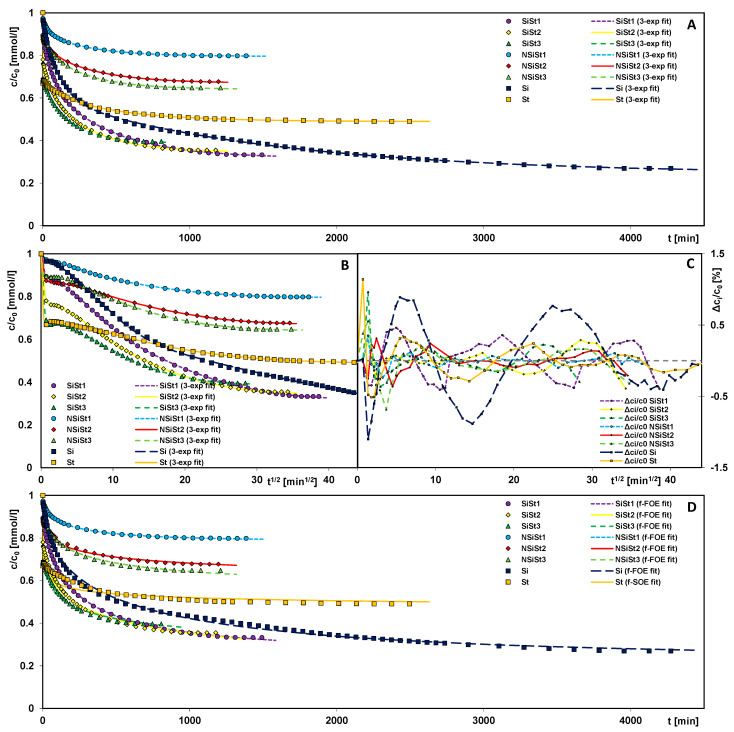

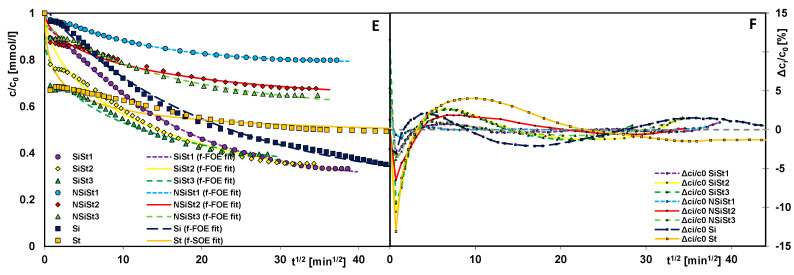
Comparison of the adsorption kinetics of methylene blue on silica–starch composites and reference materials plotted as relative adsorbate concentration versus time (**A**,**D**) and relative adsorbate concentration versus the square root of time (**B**,**E**). The lines represent fits obtained using the multi-exponential equation (**A**,**B**) and the fractal MOE equation (**D**,**E**). Panels (**C**,**F**) compare the quality of fit for individual experimental data points obtained using the multi-exponential and fractal MOE models, respectively (Δc_i_/c_0_—the difference between the relative concentration values determined based on the model and experimentally).

The adsorption kinetics were studied using the continuous recording of absorption spectra, which allowed for obtaining concentration profiles with a high density of points for the silica–starch composites (Figure 9A). To better visualize the initial and most dynamic stage of adsorption, the kinetic curves were additionally presented in a relative concentration versus the square root of time coordinate system (Figure 9B). It was observed that both the rate and efficiency of adsorption on the composites without nanosilica (the SiSt series) were higher than those obtained for materials enriched with this component (the NSiSt series).

The comparison of adsorption rates was performed based on the parameters t_20%_ and t_50%_, defined as the times required to achieve 20% and 50% decolorization in relation to the initial dye concentration, respectively. The t_20%_ values ranged from <1 to 57 min for the SiSt adsorbents and from 82 to 1046 min for the NSiSt series. The corresponding t_50%_ values for the SiSt materials were within the range of 165–549 min, whereas for the NSiSt composites these values could not be determined, as 50% decolorization was not achieved even at equilibrium. The experiments were terminated once the dye concentration stabilized, indicating the attainment of adsorption equilibrium. This stage is associated with the parameter u_eq_, representing the total uptake of the pollutant. For the SiSt1–3 composites, u_eq_ values were in the range of 0.61–0.68. In all cases, an increase in the degree of decolorization was accompanied by a prolonged time required to reach equilibrium. For the NSiSt1–3 composites, significantly lower u_eq_ values (0.20–0.36) were observed, while the equilibrium times were comparable to those of their counterparts without nanosilica.

Taking into account the combined parameters t_20%_, t_50%_, and u_eq_, the SiSt1–3 composites exhibited the highest adsorption performance. This conclusion is further supported by a comparison with the reference materials. For neat starch t_20%_ and t_50%_ values of <1 min and 1257 min were obtained, respectively, and u_eq_ was equal to 0.51. Except for the t_20%_ parameter, which was comparable to the values obtained for SiSt2 and SiSt3, the remaining kinetic parameters indicate a lower adsorption efficiency of neat starch toward the dye. For silica t_20%_ and t_50%_ values of 57 min and 549 min were obtained, respectively, and u_eq_ was equal to 0.76. This means that the adsorption process occurred much slower on silica but with better efficiency than on the SiSt1–3 composites. It was estimated that the time required to reach equilibrium was extended by as much as 2–3 times. Unfortunately, in real water treatment plants, a contact time of approximately 4000 min between the adsorbate and adsorbent may be unrealistic. Thus, the advantage of silica–starch composites as adsorbents over their pure components is evidenced by a higher amount of adsorbed pollutant relative to starch and a faster adsorption process compared with silica.

The reported kinetic parameters were determined based on optimized kinetic curves fitted using a multi-exponential equation (m-exp) (Table 3). In addition, the apparent rate constant (log k) and half-time (t_0.5_) were analyzed; however, a meaningful comparison of these parameters is only valid for systems exhibiting similar u_eq_ values. Otherwise, the assessment of adsorbent performance may be misleading. Within the SiSt series, this condition was fulfilled, allowing the adsorbents to be ranked according to their kinetic performance as follows: SiSt3 > SiSt2 > SiSt1 (log k: −2.13, −1.77, −0.18; t_0.5_: 0.46, 40, and 94 min, respectively). This order is consistent with the trends observed for the t_20%_ and t_50%_ parameters, confirming that adsorption of methylene blue on the SiSt3 composite proceeds most rapidly at all stages of the process, despite its slightly lower adsorption capacity. In contrast, the SiSt1 composite exhibits the highest adsorption capacity but is characterized by a slower dye uptake rate.

**Figure 10:**
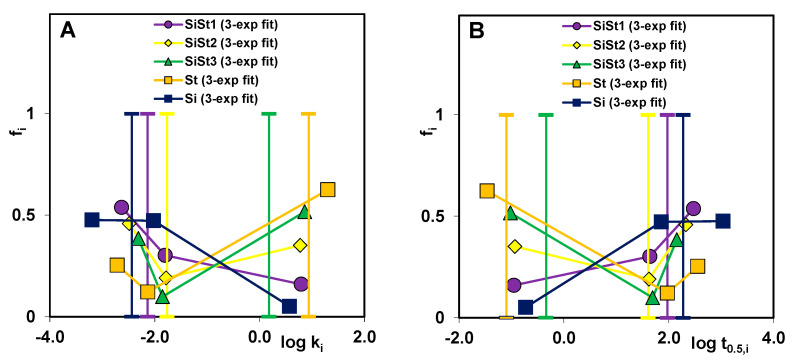

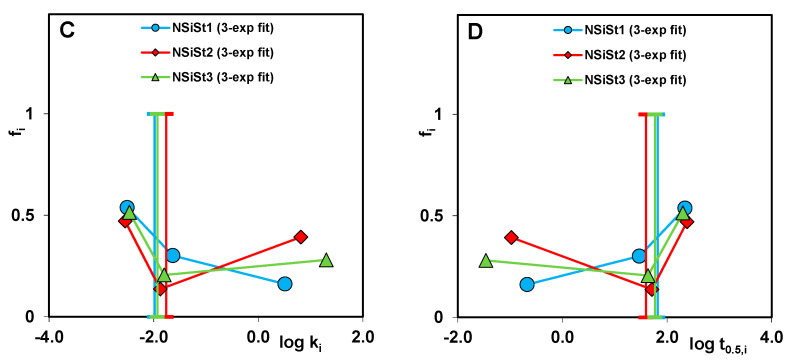
Distribution of kinetic parameters: (**A**,**C**) rate coefficients k_i_, (**B**,**D**) half-times t_1/2,i_ for three terms of the multi-exponential equation describing the adsorption of methylene blue on the silica–starch composites SiSt1–SiSt3 and reference materials (**A**,**B**), and NSiSt1–NSiSt3 (**C**,**D**).

The differences in the adsorption behavior of methylene blue on the individual composites are well illustrated by the distributions of kinetic parameters corresponding to the individual terms of the multi-exponential equation (Figure 10). These distributions involve the rate coefficients k_i_ and the associated half-times t_0.5,i_, related by the expression $t_{0.5i}=(ln\;2)/k_{i})$. Detailed data was collected in Table S1 in Supplementary Materials. The systems characterized by a higher contribution of larger k_i_ values and shorter t_0.5,i_ exhibit superior kinetic performance of adsorption. For the SiSt3, SiSt2, and SiSt1 composites, the fastest initial stage of the process accounts for the removal of 52%, 35%, and 16% of the total amount of adsorbate, respectively, whereas the slowest final stage is responsible for the removal of 38%, 46%, and 54% of the dye. The differentiation in the rates of individual adsorption stages can also be assessed based on the width of the distribution curves. A narrower range of t_0.5i_ and log k_i_ values, observed for the SiSt3 composite, indicates a lower kinetic differentiation of the process. In contrast, for the SiSt2 and SiSt1 composites, a systematic broadening of the distributions is observed, reflecting an increasing diversity of adsorption rates. The spectrum obtained for neat starch reveals a high contribution (62%) of adsorption occurring under the most favorable kinetic conditions, whereas the remaining fraction of the process proceeds at a significantly slower rate. The spectrum for silica has a different shape from the others and is significantly shifted towards parameter values indicating a slower adsorption process. The contribution of the fastest stage is negligible, amounting to 5%, while the contribution of the intermediate and slowest stages is significant, at 47% and 48%, respectively.

The distributions of kinetic parameters for the series of composites enriched with nanosilica indicate that the dye removal process proceeds most slowly on the NSiSt1 material, as evidenced by the lowest contribution of adsorption occurring during the fastest stage of the process. In contrast, the NSiSt3 composite exhibits the greatest heterogeneity of kinetic parameters, as reflected by the broadest distribution spectrum. Owing to substantial differences in the total amount of dye adsorbed by this series of materials and by the reference materials, a direct comparison of their kinetic spectra is not justified.

**Figure 11:**
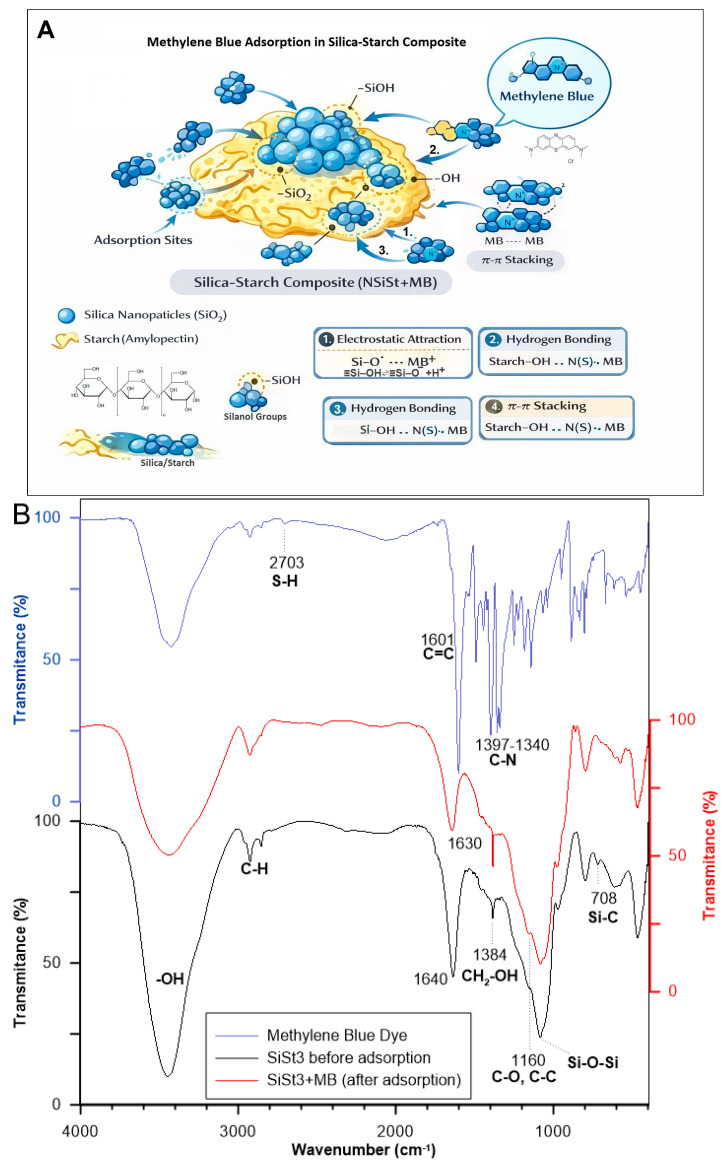
(**A**) Schematic illustration of the adsorption mechanisms of methylene blue (MB) on a silica–starch composite, (**B**) Comparison of FTIR spectra for SiST3 sample before adsorption, SiSt3+MB sample after adsorption and methylene blue.

The adsorption of methylene blue on silica–starch composites proceeds as a result of the interplay of several mechanisms (Figure 11A). The cationic nature of the dye favors electrostatic interactions with the partially negatively charged, hydrophilic surface of the composites. A key role is played by silanol groups present on the silica surface, which, upon partial dissociation, form ≡Si–O^−^ centers capable of binding dye ions. Undissociated ≡Si–OH groups, together with the abundant hydroxyl groups of starch, contribute to hydrogen bond formation, while ordered segments of the polysaccharide chains may facilitate π–π interactions. The dye removal behavior of the investigated composites can be correlated with both their chemical composition and textural properties. In the SiSt series, a systematic increase in the adsorption rate is observed with rising starch content, accompanied by a simultaneous decrease in adsorption efficiency. This effect results from the deterioration of structural parameters such as the specific surface area, total pore volume, and mesopore volume. These changes limit the accessibility of the adsorbate to adsorption sites located on the silica component surface. Neat starch does not exhibit dye-binding capabilities comparable to those of the analyzed composites. The introduction of a third component in the form of nanosilica (the NSiSt series) results in a pronounced decrease in both the adsorption efficiency and the adsorption rate of the dye. At the same time, within this series, an increase in adsorption efficiency is dependent on the nanosilica content, indicating the significant role of adsorption sites present on its surface. A correlation between the adsorption efficiency and the total pore volume of the composites was also observed.

To confirm the adsorption of methylene blue onto the surface of the starch–silica composite and the adsorbate–adsorbent interactions, FTIR spectra were analyzed for the material before adsorption (using the SiSt3 sample as an example), the pure dye, and the composite after dye adsorption (SiSt3+MB) (Figure 11B).

The spectrum of the SiSt3 composite exhibits typical bands characteristic of starch–silica materials. A broad band in the 3200–3600 cm^−1^ range corresponds to the stretching vibrations of the hydroxyl (O–H) groups present in both the starch and the silanol groups of the silica. Stretching vibrations of the C–H bonds of the polysaccharide are observed in the range of approximately 2920 cm^−1^. The most intense band in the 1000–1100 cm^−1^ region originates from asymmetric stretching vibrations of Si–O–Si bonds, which constitute the basic element of the silica structure, with a partial overlap of vibrations originating from the C–O–C and C–O glycosidic bonds of the polysaccharide (1160 cm^−1^). The methylene blue spectrum is characterized by the presence of a band at 1601 cm^−1^, corresponding to vibrations of the aromatic skeleton and the C=C and C=N bonds of the phenothiazine rings. Additionally, characteristic bands in the 1330–1400 cm^−1^ range are visible, associated with vibrations of the dimethylamino C–N groups.

After adsorption, the spectrum of the SiSt3+MB sample retains all the characteristic bands of the starch–silica composite, and a noticeable change in the intensity of the -OH hydroxyl band at ~3200 cm^−1^ is observed, becoming significantly more intense and slightly broadening. This phenomenon indicates the involvement of starch hydroxyl groups and the silanol groups of silica in interactions with methylene blue molecules, likely through the formation of hydrogen bonds. The band at 1640 cm^−1^ (related to -OH bending vibration due to the highly hydrophilic nature of the biopolymer structure) shifts toward lower wavenumbers (to 1630 cm^−1^) after dye adsorption (for the dye, a signal corresponding to the vibration of the aromatic skeleton at 1601 cm^−1^ appears in this range). Furthermore, a signal associated with the polysaccharide CH_2_-OH side chain group appears due to the intense signals of the C-N groups in this range. The formation of hydrogen bonds between the hydroxyl groups of the support and the C-S groups of the dye can also be confirmed by the disappearance of the signal characteristic for this group in the material after adsorption, i.e., at the 2703 cm^−1^ position. A disappearance of the signal at the 708 cm^−1^ position was also observed, which may be related to the weakening of the vibrational capacity of the Si-C group due to binding to the dye molecule.

Kinetic data were analyzed using a multi-exponential (m-exp) equation and a fractal MOE equation (f-MOE). The determined kinetic parameters and the quality of fitting are summarized in Table 3. The multi-exponential equation describes adsorption within a compartmental model involving parallel and/or consecutive first-order processes. The fractal MOE equation represents a modification of the classical MOE model through the introduction of an additional parameter, the fractality coefficient (*p*), which reflects the heterogeneity of the adsorption system. The greater the deviation of *p* from unity, the broader the distribution of rate coefficients describing the kinetics. For the analyzed systems, *p*-values ranged from 0.30 to 0.58, indicating a substantial heterogeneity of rate coefficients. During the optimization procedure using the f-MOE equation, the parameter f_2_ takes values of 0 or 1, leading to a simplification of the equation to either the fractal first-order (f-FOE) or fractal second-order (f-SOE) form. A comparison of the theoretical curves and their agreement with the experimental data for the m-exp model (Figure 9C) and the f-MOE model (Figure 9F) reveals lower flexibility of the fractal approach, particularly in the initial stage of the experiment. The inferior accuracy of the kinetic description using the f-MOE equation is further confirmed by higher values of the goodness-of-fit indicators 1 − R^2^ and SD(c)/c_0_.

Based on the methylene blue adsorption values determined for the silica–starch composites (Table 3), it can be observed that the adsorption is relatively low (expressed in µmol/g). However, it should be emphasized that these values are strongly dependent on the experimental conditions employed and therefore should not be interpreted as adsorption capacity. A more reliable measure of adsorption capacity should be derived from equilibrium adsorption isotherms. Nevertheless, silica–starch composites represent an attractive class of adsorbent materials in the context of sustainable development and green chemistry. The evaluation of adsorbent suitability should consider not only adsorption performance but also the environmental and economic impacts associated with material synthesis, application, and end-of-life management. Silica–starch composites can be prepared from abundant and readily available raw materials using cost-effective and relatively simple methods, making them promising alternatives to more efficient yet often less sustainable adsorbents, such as extensively modified silica-based materials and synthetic polymeric adsorbents.

### 2.3. Visualization of Dye Adsorption on Silica–Starch Composites

By recording IR spectra at each point within a defined region (FTIR mapping), it is possible to visualize the spatial distribution of functional groups and molecular components, such as dye molecules, on the adsorbent surface, providing insights into how and where molecules are incorporated into the intergranular network. In the case of silica–starch materials, direct visualization of the distribution of silica and starch within the material at the microstructural level was achieved (exemplified by NSiSt1), as well as the distribution of silica, starch, and dye components within the material after dye adsorption (NSiSt1+MB).

**Figure 12:**
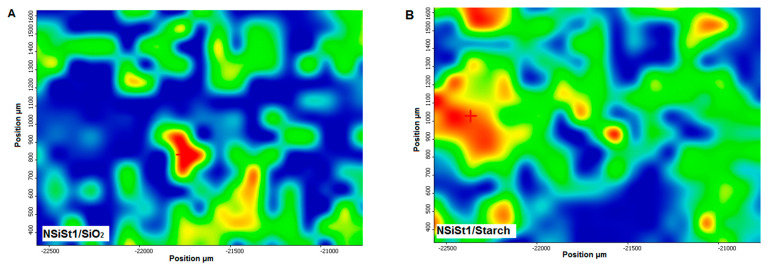

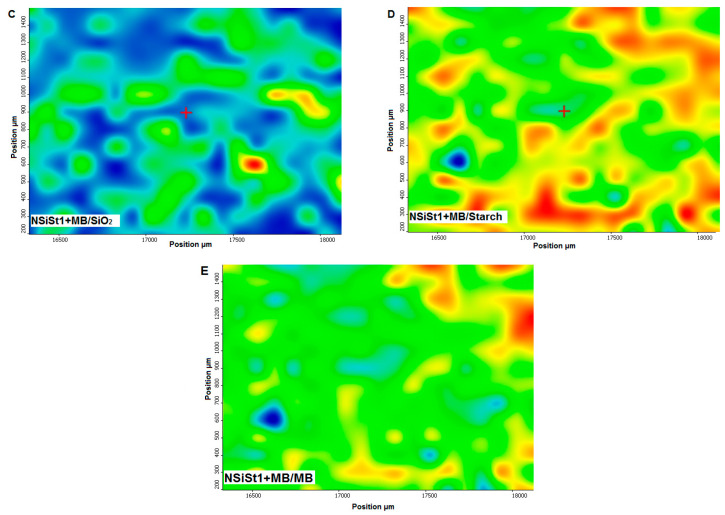
Correlation map of the components on NSiSt1 and NSiSt1/methylene blue system by FTIR mapping: (**A**) FTIR map of the 2 µm mapped area of NSiSt1 sample as a distribution correlation map of silica spectra (generated from the point marked with a red cross and presented in Figure S1 in Supplementary Materials File), (**B**) FTIR mapp of starch component of NSiSt1 sample; (**C**) FTIR map of the distribution correlation map of silica, (**D**) starch, and (**E**) methylene blue in relation to NSiSt1+MB. Signal intensity is presented on a color scale from 0 (blue—no or very low intensity) to 1 (red—highest intensity).

Figure 12A,B show the spatial distribution of the silica and starch signals within the analyzed material region. The signal intensity distribution is heterogeneous, indicating local differences in the silica phase concentration. The image is dominated by areas of low signal intensity (blue), corresponding to silica-poor regions, interspersed with distinct zones of increased intensity (green–red). In contrast to the silica map in Figure 12B, a greater proportion of areas of medium and high signal intensity (green–red) is observed, indicating that starch remains somewhat more uniformly distributed within the grains. At the same time, the absence of large, continuous zones of very high intensity for both components indicates that silica and starch aggregation is limited, and the particles remain relatively well dispersed within the material structure. The starch signal distribution is more extensive and exhibits greater spatial continuity than that of silica. In many places, areas of high starch intensity correspond to regions of lower silica signal intensity, suggesting a complementary distribution of both phases. The lack of distinct phase segregation at the microscale indicates that the composite components are well mixed and interpenetrated within the material structure. At the same time, the presence of local areas of increased silica signal intensity suggests partial aggregation of SiO_2_ particles, which is typical for systems containing silica nanoparticles. Figure 12C–E present FTIR maps illustrating the spatial distribution of components in the silica–starch composite after the adsorption of methylene dye.

The methylene dye distribution map shows a heterogeneous distribution of dye molecules within the material. Areas of increased signal intensity indicate local sites of preferential adsorption. Spatial analysis suggests that MB molecules are adsorbed both in regions associated with the starch phase and near the silica domains.

This distribution indicates that the adsorption process occurs at different types of active sites, including both the hydroxyl groups of the starch and the silanol groups on the silica surface. At the same time, the lack of clear segregation of the dye into a single domain suggests that MB molecules can penetrate the porous intergranular network of the composite, where they interact with both components of the material. Methylene blue molecules are distributed throughout the composite structure, with the observed local intensity maxima indicating the presence of preferential adsorption sites associated with the silica surface and the starch polysaccharide network.

### 2.4. Reusability Tests

**Figure 13:**
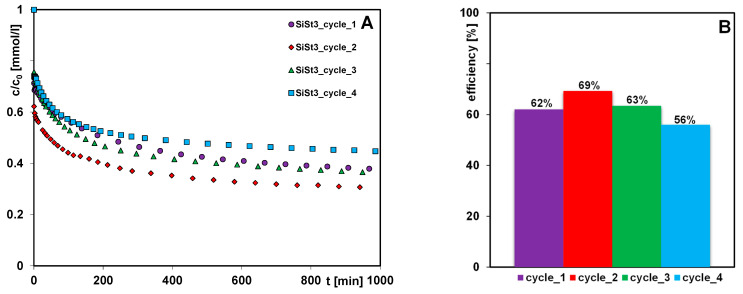
Adsorption profile of methylene blue as a function of time (**A**) and reusability of the SiSt3 composite over four consecutive adsorption–desorption cycles (**B**).

The operational stability of the selected adsorbent (SiSt3) from the SiSt series was evaluated over four consecutive adsorption–desorption cycles using ethanol as the desorbing agent (Figure 13A,B). The material maintained a high adsorption efficiency throughout all regeneration cycles (56–69%), demonstrating operational stability and a high adsorption capacity after repeated reuse. The noticeable improvement in the adsorption kinetics and the increase in dye removal efficiency observed in the second cycle can likely be attributed to the desorption treatment with ethanol, which not only removed the adsorbed dye molecules but also extracted residual organic species remaining from the synthesis process. Due to the presence of starch, during the synthesis of the silica–starch composites, conventional calcination was replaced by washing with a water–ethanol mixture. This procedure did not provide for the complete removal of organic residues (surfactant and unreacted tetraethyl orthosilicate), which could partially block the adsorption sites during the first adsorption cycle. In the third cycle, the course of dye adsorption was similar to that on the initial composite, although the efficiency was still higher. In the last cycle, a slight decrease in the adsorption efficiency was observed, which ultimately led to the conclusion that regeneration with the used eluent initially improved the adsorption efficiency, but in the subsequent cycles, it gradually deteriorated due to the incomplete desorption of the adsorbate and therefore the presence of the dye from the previous cycle on the adsorbent surface.

## 3. Materials and Methods

### 3.1. Chemicals and Materials

#### 3.1.1. Chemicals

Starch soluble (from potato) was purchased from Sigma Aldrich (Poznań, Poland). Hydrochloric acid (35–38 wt%) and Ethanol (96%) were supplied by POCh (Poznań, Poland), and tetraethyl orthosilicate (TEOS, ≥99.0%) was purchased from Sigma-Aldrich (Poznań, Poland). The nanosilica phase, in the form of fumed silica materials with a particle size of 0.2 µm marked as NS, was obtained from Sigma-Aldrich (Poznań, Poland). Methylene blue, a cationic dye was purchased from Avantor Performance Materials Poland S.A. (Gliwice, Poland).

#### 3.1.2. Synthesis of Starch–Silica Composites

Starch–silica composites were synthesized using a modified sol–gel approach and divided into two material groups differing in the composition of the starch and silica phase, respectively (Table 4). In both cases, tetraethyl orthosilicate (TEOS) was used as the silica precursor, and the syntheses were conducted in an acidic medium. In the synthesis of the first material group, specific amounts of starch were weighed into three reaction bottles containing 50 mL of distilled water. The contents of the reaction bottles were heated to 45 °C with vigorous stirring for approximately 30 min. Then, 10 mL of ethanol was added dropwise to the reaction solutions. Stirring was continued for 10 min, after which the reaction medium was acidified by adding 30 mL of 2M hydrochloric acid. Next, 10 mL of TEOS, which acted as the silica precursor, was added to the prepared solutions. The addition of TEOS initiated hydrolysis and condensation reactions, leading to the formation of a three-dimensional Si–O–Si network in the presence of the polysaccharide. The mixture was kept under constant stirring for 24 h at 40 °C. To stabilize the porous structure, the mixture was aged under hydrothermal conditions. The resulting suspension was transferred to a sealed reaction vessel and incubated at 70 °C for another 72 h without stirring. After the aging process was completed, the precipitate was separated by filtration, washed several times with distilled water and ethanol, and dried in an air atmosphere at 60 °C for 12 h. The resulting materials were obtained as white solids and labeled as SiSt1, SiSt2, and SiSt3, corresponding to the increasing starch content. For the second group of materials, 50 mL of distilled water was placed in a reaction vessel, followed by the addition of 500 mg of starch and nanosilica in amounts of 50, 100, or 500 mg. The mixtures were heated to 45 °C and stirred for 30 min. Subsequently, 30 mL of 2 M hydrochloric acid was added to acidify the system. Next, 10 mL of TEOS was added to the reaction mixtures. The suspensions were stirred at 40 °C for 24 h and then aged under hydrothermal conditions at 70 °C for 72 h without stirring. The resulting solids were isolated by filtration, thoroughly washed with distilled water and ethanol, and dried at 60 °C for 12 h. The obtained white powders were denoted as NSiSt1, NSiSt2, and NSiSt3, depending on the colloidal silica content.

### 3.2. Textural, Structural, Morphological and Thermal Characterization of Starch–Silica Composites

The porous structure of the starch–silica materials was characterized by nitrogen adsorption–desorption measurements at 77 K using an ASAP 2020 analyzer (Micromeritics, Norcross, GA, USA). The measurements were carried out over a relative pressure range (*p*/*p*_0_) from 0 to 0.97. The specific surface area (S_BET_) was calculated from the adsorption isotherm according to the Brunauer–Emmett–Teller (BET) method. The pore size distributions were determined from the adsorption branches of the isotherms using the DFT method and the Barrett–Joyner–Halenda (BJH) model assuming a cylindrical pore geometry with the KJS correction [101]. The total pore volume (V_t_) was estimated from the amount of nitrogen adsorbed at *p*/*p*_0_ = 0.97. The external surface area (S_ext_) and the mesopore volume (V_p_) were determined by the α_s_ method using a silica standard sample [102]. Moreover, to determine the micropore distribution functions, the Horvath–Kawazoe method was applied. Before analysis, the powdered samples were degassed at 60 °C under vacuum (1 mmHg) for 24 h in the degassing port of the analyzer.

Small-angle X-ray scattering (SAXS) measurements were performed using an Empyrean diffractometer (PANalytical, Malvern, UK) equipped with a Cu anode X-ray tube and a SAXS/WAXS sample stage operating in capillary mode. The instrument was powered by a 4 kW high-voltage generator operated at 40 kV and 40 mA. The incident X-ray beam was conditioned using a W/Si graded elliptical mirror. Scattering data were collected over a 2θ range from −0.1° to 4.0°, with a step size of 0.005° and a counting time of 1.76 s per step, yielding approximately 800 data points per scan. A 0.2 mm Cu beam attenuator was applied for measurements in the vicinity of the primary beam. The scattered X-rays were recorded using a PIXcel3D detector through a receiving slit with an active length of 0.05 mm. The magnitude of the scattering vector, q, was calculated according to the relation q = 4π sin θ/λ, where θ is the scattering angle and λ = 1.5418 Å corresponds to the wavelength of Cu Kα radiation. Background scattering was determined from air measurements using an empty capillary holder and was subtracted from the sample scattering data.

The surface morphology of the samples was examined using field-emission scanning electron microscopy (FE-SEM) with a Quanta™ 3D FEG microscope (FEI Company, Hillsboro, OR, USA) operated at an accelerating voltage of 30 kV.

Chemical mapping was performed using a Nicolet iN10 MX FTIR microscope equipped with a motorized XY stage and a liquid-nitrogen-cooled mercury cadmium telluride (MCT) detector. Measurements were conducted in reflection mode. Chemical distribution maps were generated and processed using OMNIC Specta™ software (version 8.1) based on spectral correlation with reference spectra of the silica carrier and the pure dye (Figure S1). Transmission FTIR spectra were obtained using a FTIRNicolet 8700A spectrometer (Thermo Scientific, Waltham, MA, USA).

Thermal analysis was performed using a STA 449 Jupiter F1 instrument (NETZSCH, Selb, Bavaria, Germany). Approximately 12 mg of each sample was heated at a rate of 10 °C·min^−1^ over a temperature range of 30–1000 °C under a flow of synthetic air (50 mL·min^−1^). Measurements were carried out in alumina (Al_2_O_3_) crucibles using a TG–DSC setup equipped with a type S thermocouple. An empty Al_2_O_3_ crucible was used as a reference. Data acquisition and processing were performed using NETZSCH Proteus^®^ software (version 6.1).

### 3.3. Dye Adsorption Kinetic Measurements

Kinetic studies were carried out using the continuous registration method of the adsorbate solution spectra [103,104]. The experiments were conducted in a glass vessel maintained at 25 °C, into which 0.2 g of the adsorbent and 50 mL of a dye solution with a concentration of 0.04716 mmol/L were introduced. At specified time intervals, samples were automatically withdrawn from the system and directed to a flow-through cuvette, where UV-Vis spectra were recorded using a Cary 100 spectrophotometer (Varian Inc., Melbourne, VIC, Australia). Throughout the entire experiment, the suspension was subjected to continuous mechanical stirring at a speed of 120 rpm. Changes in absorbance at the maximum of the characteristic absorption band of the dye were converted into changes in its concentration as a function of time. The obtained data were further analyzed using: (i) the multi-exponential equation (m-exp) [105]:

(1) $$c=\left(c_{0}-c_{eq}\right)\sum_{i=1}^{n}f_{i}exp\left(-k_{i}t\right)+c_{eq}$$

(2) $$c=c_{0}-c_{0}u_{eq}\sum_{i=1}^{n}f_{i}\left[1-exp\left(-k_{i}t\right)\right]\;$$

where “*i*” is the term of the m-exp equation, *k_i_* is the rate coefficient, and u_eq_ = 1 − c_eq_/c_0_ is the relative loss of adsorbate from the solution.

(ii) fractal-like MOE equation (f-MOE):

(3) $$F=\frac{{1-exp\left(-k_{1}t\right)}^{p}}{{1-f_{2}exp\left(-k_{1}t\right)}^{p}}$$

where *p* is the fractal coefficient [106,107,108,109].

## 4. Conclusions

Analysis of the Dv(R) function indicates a hierarchical structural organization of the silica–starch composites, comprising primary silica nanostructures (~3–4 nm), intermediate structures (~8–10 nm) related to starch organization, and larger mesostructural aggregates (~18 nm and above). With increasing starch content, a gradual reorganization of the material’s mesostructure and a change in the size distribution of heterogeneities are observed.

TG/DTG/DSC analysis showed that starch and silica–starch composites exhibit a similar course of thermal degradation, and the characteristic transition temperatures remain comparable for all investigated materials. The successive stages of degradation include: the removal of physically adsorbed water (below 150 °C), the initial depolymerization associated with the cleavage of α-(1 → 4) bonds and intramolecular dehydration reactions (150–270 °C), the decomposition of the polysaccharide structure combined with oxidation processes (260–400 °C), as well as the combustion of the residual carbonaceous matter (400–560 °C). The presence of silica in the composites increases the mass residue at high temperatures, which results from the presence of a stable inorganic phase.

Silica–starch composites demonstrate the ability to effectively remove methylene blue from aqueous solutions, and their adsorption properties depend both on the material composition and its textural parameters. The absence of the nanosilica component in the composite results in a faster adsorption process and higher decolorization efficiency of the solution. In turn, increasing the content of the starch component relative to the silica gel enhances the adsorption rate, but at the expense of reduced dye removal efficiency. This effect arises from the deterioration of structural parameters such as the specific surface area, total pore volume, and mesopore volume. These changes limit the access of the adsorbate to adsorption sites located on the surface of the silica component. The observed multi-step adsorption kinetics, described by the multi-exponential (m-exp) equation and the fractal MOE (f-MOE) equation, indicate a diversity in the energy and accessibility of adsorption sites, which is a consequence of the heterogeneous structure of the composites.

Silica–starch composites exhibit a mesoporous structure that governs both the accessibility of adsorption sites and the overall dye removal performance. The adsorption of methylene blue proceeds via a combination of electrostatic interactions between the cationic dye and negatively charged silanol groups (≡Si–O^−^), hydrogen bonding involving ≡Si–OH and starch hydroxyl groups, and possible π–π interactions with ordered polysaccharide segments. The adsorption mechanisms were also confirmed by FTIR spectra analysis of the silica–starch composite before and after adsorption and of the pure dye. The process is strongly influenced by mass transport, including diffusion to the surface and within the porous network, leading to multi-step adsorption kinetics and heterogeneous site accessibility.

FTIR mapping confirms that the NSiSt1 material is characterized by a microstructurally heterogeneous distribution of components. It also confirms that in the NSiSt1+MB composite, the dye molecules are effectively incorporated into the material structure and distributed within the intergranular network of the composite. Furthermore, the presence of numerous silica–starch interfaces promotes the formation of diverse adsorption sites, which may be responsible for the material’s high adsorption capacity for the methylene dye.

Regeneration and reusability tests demonstrated good adsorption efficiency over four regeneration cycles (56–69%), confirming the operational stability and a high adsorption capacity after repeated use. Studies will continue using other eluents and regeneration methods.

## Figures and Tables

**Table 1:** The textural parameters of starch–silica composites.

Composite	S_BET_ ^1^ [m^2^/g]	S_ext_ ^2^ [m^2^/g]	S_mic_ ^3^ [m^2^/g]	V_t_ ^4^ [cm^3^/g]	V_mic_ ^5^ [cm^3^/g]	V_p_ ^6^ [cm^3^/g]	V_p_/V_t_ ^7^	D_BJH ads_ ^8^ [nm]	D_mo_ H-K ^9^ [nm]
SiSt1	676	4	128	0.41	0.05	0.39	0.95	2.71	1.07
SiSt2	550	2	151	0.30	0.06	0.29	0.97	2.40	0.99
SiSt3	455	2	93	0.26	0.04	0.25	0.96	2.50	1.22
NSiSt1	277	3	-	0.21	-	0.21	1	3.17	1.50
NSiSt2	439	14	32	0.27	0.01	0.25	0.93	2.78	1.25
NSiSt3	360	4	20	0.28	-	0.27	0.96	3.31	1.45

^1^ BET surface area of starch–silica composites, using experimental points at a relative pressure of *p*/*p*_0_ ~0.035–0.31, where *p* and *p*_0_ are denoted as the equilibrium and saturation pressure of nitrogen. ^2^ External surface area determined from α_s_ plot. ^3^ Micropore surface area calculated from t-plot. ^4^ Total pore volume calculated from the amount of nitrogen adsorbed at *p*/*p*_0_ = 0.99. ^5^ Micropore volume calculated from t-plot. ^6^ Primary mesopore volume determined from α_s_ plot. ^7^ Mesopore contribution. ^8^ The pore diameter estimated from PSD maximum of BJH theory from the adsorption data. ^9^ The micropore diameter estimated from HK theory.

**Table 2:** Thermal behavior of neat starch and composites (DTG/DSC T_max_ is the maximum peak value determined for DTG or DSC curves, ΔH—specific enthalpy of the process).

Adsorbent	Temperature Range [°C]	Mass Loss [%]	Total Mass Change [%]/Starch Content [%]	DTG T_max_ [°C]	DSC T_max_ [°C] (Endo ↑; Exo ↓)	ΔH_exo_ [J/g]
SiSt1	35–157	2.73	19.01/20.47	88	95 ↑	−927
	157–270	3.25		226	222 ↑	
	270–420	6.24		335	347 ↓	
	420–563	5.35		507	502 ↓	
SiSt2	35–153	1.94	22.21/23.91	88	101 ↑	−1154
	153–270	4.26		228	224 ↑	
	270–409	7.59		332	346 ↓	
	409–557	6.84		505	499 ↓	
SiSt3	35–141	2.36	36.59/39.35	88	101 ↑	−2062
	141–269	7.68		228	225 ↑	
	269–408	13.33		332	349 ↓	
	408–561	11.73		503	502 ↓	
NSiSt1	35–191	5.36	33.53/36.10	88, 148	113, 155 ↑	−1778
	191–273	5.12		233	226 ↑	
	273–423	12.64		331	350 ↓	
	423–572	9.11		497	497 ↓	
NSiSt2	35–183	3.89	33.19/35.73	81, 158	101, 162 ↑	−1833
	183–263	5.62		224	218 ↑	
	263–241	13.16		330	348 ↓	
	241–575	3.09		501	496 ↓	
NSiSt3	35–183	3.42	31.75/34.18	80, 159	109, 163 ↑	−1731
	183–270	5.84		225	220 ↑	
	270–421	12.19		328	347 ↓	
	421–558	8.78		499	498 ↓	
St	35–136	3.23	92.89/100	80	101 ↑	−5410
	136–272	23.64		230	228 ↑	
	272–395	29.93		325	353 ↓	
	395–559	34.24		500	493 ↓	

**Table 3:** Comparison of kinetic parameters determined based on the multi-exponential equation (m-exp) and the fractal MOE equation (f-MOE).

Adsorbent	Fit	f_2_/*p*	t_20%_/t_50%_ [min]	log k_1_	t_0.5_ [min]	a_eq_/u_eq_	SD(c/c_0_) [%]	1 − R^2^
SiSt1	3-exp	-	25/300	−2.13	94	80/0.68	0.28	1.56 × 10^−4^
	f-FOE	0/0.58	23/300	−2.40	133	84/0.70	1.15	2.85 × 10^−3^
SiSt2	3-exp	-	0.35/208	−1.77	40	77/0.66	0.16	7.43 × 10^−5^
	f-FOE	0/0.30	3.78/208	−2.52	98	101/0.86	2.72	2.61 × 10^−2^
SiSt3	3-exp	-	0.15/165	0.18	0.46	72/0.61	0.26	3.20 × 10^−4^
	f-FOE	0/0.30	0.26/158	−2.26	54	87/0.71	3.53	6.72 × 10^−2^
NSiSt1	3-exp	-	1046/-	−1.98	66	24/0.20	0.10	1.88 × 10^−4^
	f-FOE	0/0.55	1000/-	−2.24	89	25/0.20	0.35	2.65 × 10^−3^
NSiSt2	3-exp	-	94/-	−1.76	40	39/0.33	0.17	2.95 × 10^−4^
	f-FOE	0/0.30	62/-	−2.31	61	47/0.40	1.92	4.72 × 10^−2^
NSiSt3	3-exp	-	82/-	−1.93	59	42/0.36	0.25	4.58 × 10^−4^
	f-FOE	0/0.47	75/-	−2.46	132	49/0.39	1.43	1.76 × 10^−2^
St	3-exp	-	0.05/1257	0.93	0.08	56/0.51	0.27	7.18 × 10^−4^
	f-SOE	1/0.30	0.48/2511	−0.60	4	67/0.57	2.99	9.65 × 10^−2^
Si	3-exp	-	57/549	−2.44	189	89/0.76	0.46	3.75 × 10^−4^
	f-SOE	0/0.48	49/549	−2.68	222	90/0.78	1.23	2.85 × 10^−3^

**Table 4:** Name and constituent components of the samples.

Sample	The Starch Amount in the Reaction Mixture [mg]	Nanosilica Amount [mg]
SiSt1	50	-
SiSt2	100	-
SiSt3	500	-
NSiSt1	500	50
NSiSt2	500	100
NSiSt3	500	500

## Data Availability

The data presented in this study are available upon request from the corresponding authors.

## Supplementary Materials

The following supporting information can be downloaded at: https://www.mdpi.com/article/10.3390/ijms27146243/s1.
