# Supplementary material for: Mechanistic Insights into Biodegradable Silica–Starch Composite Materials—Structural and Adsorption Properties
Source: Int J Mol Sci. 2026 Jul 14;27(14):6243. doi: 10.3390/ijms27146243 (PMC13410077; doi:10.3390/ijms27146243)
Supplement: Supplementary file 1 [file ijms-27-06243-s001.zip › ijms-4385371-supplementary.pdf]

Supplementary Materials

Physicochemical characterization and dye adsorption performance of silica–starch composite materials

Malgorzata Zienkiewicz-Strzalka[1,*], Magdalena Blachnio[1], Krystian Czuchryta[1] and Anna Derylo-Marczewska [1,*]

[1] Department of Physical Chemistry, Institute of Chemical Sciences, Maria Curie-Sklodowska University,
Maria Curie-Sklodowska Square 3, 20-031 Lublin, Poland; magdalena.blachnio@mail.umcs.pl (MB), krystiancz02@onet.pl (KCz)

* Correspondence: anna.derylo-marczewska@mail.umcs.pl (A.D.-M.); malgorzata.zienkiewicz-strzalka@mail.umcs.pl (M.Z.-S.); Tel.: +48-0815375637

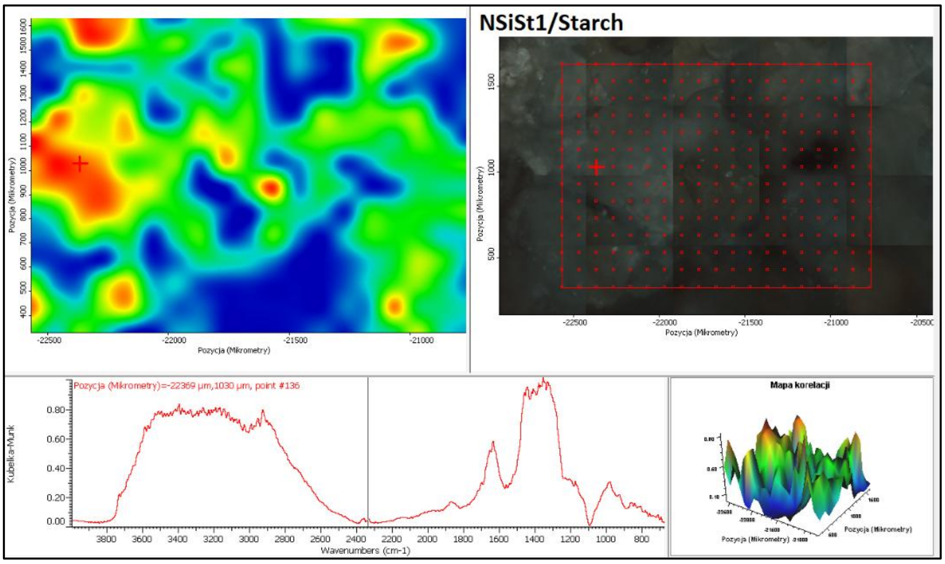

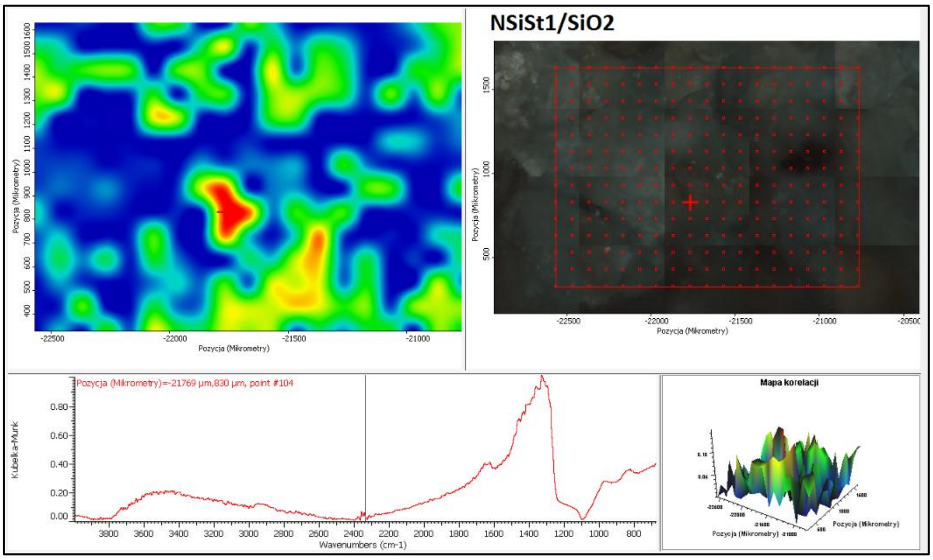

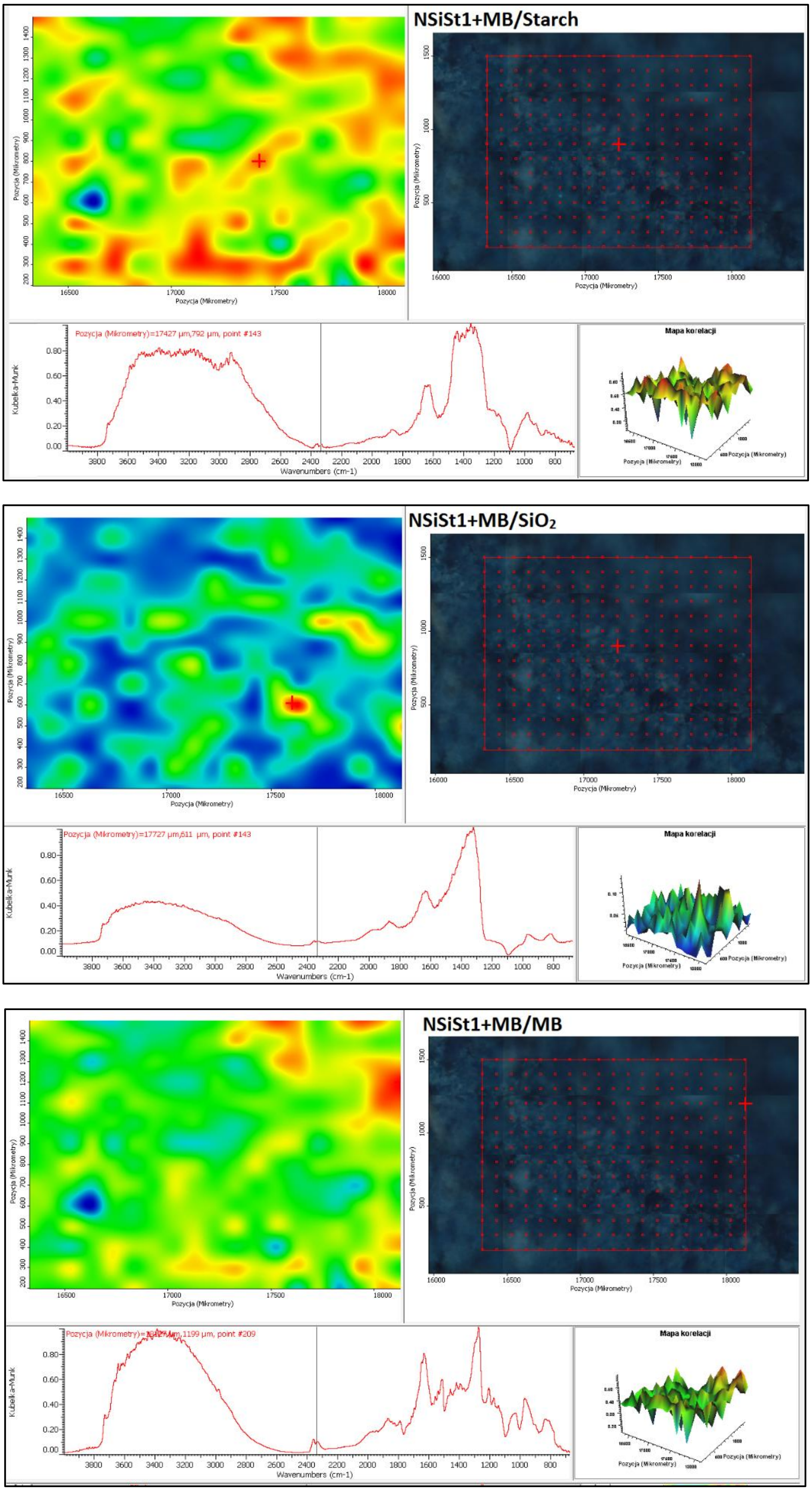

Figure S1. Correlation map of the components and FTIR spectra generated from point marked in FTIR map.

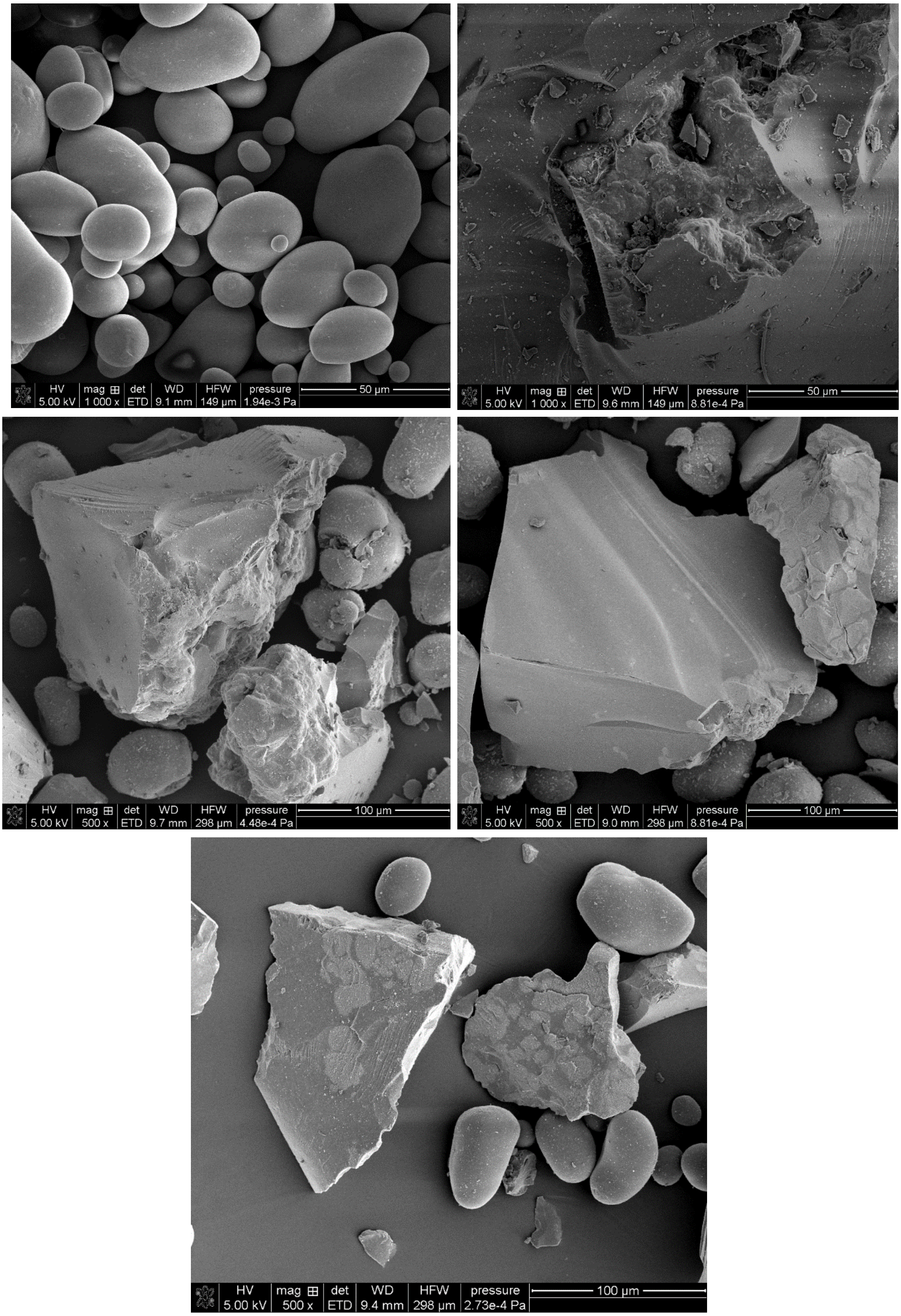

Figure S2. Scanning electron microscopy (SEM) images illustrating the morphology of the investigated starch and silica-starch materials. (A) SEM image

of starch granules with predominantly oval and spherical shapes, (B) SEMimage of SiSt1 sample, (C) SiSt3, (D) NSiSt1 and (E) NSiSt3.

Table S1. Parameters of the multiexponential equation for methylene blue adsorption on silica-starch composites.

**Adsorbent**	**i**	**f_i**	**log k_i**	**log $t_{0.5,i}$**
SiSt1	1	0.16	0.79	-0.95
	2	0.30	-1.80	1.64
	3	0.54	-2.63	2.47
SiSt2	1	0.35	0.77	-0.93
	2	0.19	-1.78	1.63
	3	0.46	-2.49	2.33
SiSt3	1	0.52	0.86	-1.02
	2	0.10	-1.85	1.69
	3	0.38	-2.31	2.15
NSiSt1	1	0.16	0.52	-0.67
	2	0.30	-1.63	1.47
	3	0.54	-2.50	2.34
NSiSt2	1	0.39	0.82	-0.98
	2	0.14	-1.87	1.71
	3	0.47	-2.54	2.38
NSiSt3	1	0.28	1.30	-1.46
	2	0.21	-1.80	1.64
	3	0.51	-2.46	2.30
St	1	0.62	1.30	-1.46
	2	0.12	-2.13	1.97
	3	0.25	-2.71	2.56
